# Characteristics and perceptions of twice-weekly webinars for primary care physicians in Japan: a qualitative study

**DOI:** 10.5116/ijme.5b6b.21e1

**Published:** 2018-08-31

**Authors:** Shinji Kimura, Hirotaka Onishi, Minori Kawamata

**Affiliations:** 1 Department of English, Center for Medical Education, Sapporo Medical University, Japan; 2 International Research Center for Medical Education, University of Tokyo Graduate School of Medicine, Japan; 3 Department of Psychiatry, Tenshi Hospital, Japan

**Keywords:** Continuing medical education, distance education, interactive learning, video conference, program evaluation, Japan

## Abstract

**Objectives:**

To explore the characteristic features and perceived value of free twice-weekly webinars predominantly focusing on the continuing professional development of primary care physicians in Japan.

**Methods:**

In this qualitative study, we conducted a focus group of the webinars’ participants (n=6, a purposive sample). The discussion was recorded, with the recording subsequently transcribed, separated into meaningful segments and then open-coded until thematic saturation was reached. Concepts were generated through selective coding. Finally, the extracted concepts were grouped into categories.

**Results:**

Extracted concepts were grouped into five categories: technological breakthroughs, the creation of learning opportunities, external interaction, stimulation of internal interaction, and the advantages and disadvantages of nationwide expansion. The webinars were perceived to provide a comfortable learning climate, enabling physicians to teach one another, share their experiences and become virtual colleagues. The chat system stimulated real-time interaction between both a main speaker and participants and the participants. Participants were able to ask questions or give comments in a stress-free atmosphere. The webinars were found to elicit real-time, internal interaction within participating sites without interrupting the sessions. Participants also highly valued the absence of commercial sponsorship. The expansion of the webinars raised two concerns: the possibility of speakers becoming nervous and the increased burden on the organizers.

**Conclusions:**

The webinars have successfully allowed sharing of unbiased information and experiences in a comfortable, multifaceted interactive learning environment, enabling participants to feel connected. The chat system permitted interaction not feasible in face-to-face learning opportunities and has shown great promise as a means of online medical education.

## Introduction

Continuing professional development (CPD) is an essential component of medicine that keeps physicians and other professionals up-to-date. Rural physicians have limited access to CPD opportunities compared to those in urban areas due to time, expense, travel issues, and a lack of coverage in their practices.^1^^,^^2^ Videoconferencing (real-time two-way audio plus video communication between two or more locations) has been utilized in CPD since the late 1990s.^3^^-^^7^ In the initial phase, videoconferencing was performed on a telephone line that was called an Integrated Services Digital Network (ISDN), which was a digital network that had a higher speed than the traditional telephone network.^8^ High-speed Internet connections became widely available in the early 2000s, and Internet videoconferencing emerged as a useful tool for expanding access to CPD for healthcare professionals who sought real-time, two-way interaction.^1^^,^^9^

Rural physicians suffer from professional isolation. In Japan, many rural physicians work alone in their clinics. They experience anxiety about seeing patients who are beyond the scope of their specialty training, psychological pressure stemming from making clinical decisions by themselves, a lack of locum physicians and a sense of loneliness.^10^ They also have difficulty improving their knowledge, skills, and level of practice. In Australia and Canada, to address this situation, there are online CPD programs that are tailored to rural physicians.^11^^,^^12^

In Japan, Internet videoconferences have been held for CPD by small groups of primary care physicians since around 1999.^13^^-^^15^ Online CPD on a much larger scale has been provided by the Japan Medical Association (JMA), professional societies and pharmaceutical companies.^16^^-^^19^ Those that were created by JMA and professional societies are videos on demand (VOD) and lack real-time interaction. Pharmaceutical companies offer webinars that are usually one-way videos and voice with text chat interaction. Many, if not all, studies of exposure to information from pharmaceutical companies have shown an association with higher prescribing frequency, higher costs or lower prescribing frequency and thus such exposure has been discouraged.^20^

For over ten years, the Department of General and Community Medicine at Sapporo Medical University has conducted webinars for continuing professional development (CPD) of (mainly) primary care physicians based in Japan. The present report is a qualitative study of the characteristics of these webinars and how participants view them. Improving access to CPD, including videoconferencing, has been shown to reduce a common feeling of professional isolation through connecting participants.^1^^,^^6^^,^^21^^,^^22^ Qualitative studies have shown that participants in online CPD highly value interpersonal interaction.^23^ Interaction during videoconferences or webinars has been known to occur via voice and/or by text chats.^5^^,^^24^ In CPD videoconferences or webinars intended primarily for physicians, vocal interaction among learners has been reported, but no study has thus far reported on an extensive use of chat tools or their effect or usefulness in interaction.^25^ There is also a lack of research on how webinars for primary care physicians are conducted over an extended period of time. This is the first such study. This study has two objectives: 1) explore how the webinars, geared mainly toward primary care physicians, have gauged their usefulness and 2) examine the webinars’ characteristic features, including the usage of the chat system.

This study examines two webinars for CPD that have been held since 2004 by the Department of Community and General Medicine, Sapporo Medical University School of Medicine, in Hokkaido, Japan. (Although our sessions have characteristics of both videoconferences and webinars, from their onset they have been referred to as “webinars” and for the purpose of simplicity, which will be their designation hereafter in this manuscript.) The webinars are especially for primary care physicians, particularly those in rural settings. However, they are also attended by primary care physicians in metropolitan, urban and remote areas throughout Japan, as well as some specialists and other health professionals. They are conducted twice a week. One webinar, which is held on Wednesdays, is called the Primary Care Conference (PCC) and is either a case presentation/discussion or a journal club. The other webinar meets on Thursdays and is called the Primary Care Lecture Series (PCLS). This is a didactic lecture followed by questions and answers. For both webinars, participating sites log onto the designated website by using their IDs and passwords. There is a moderator who appears at the beginning and the end of the session; the moderator’s main roles are to introduce the speaker and elicit questions. A video of the speaker, as well as slides, is then shown on the computer screen and the voice of the speaker is heard from the personal computer. A chat system is incorporated into the system by which participants can ask questions and give comments during the session. The speaker answers them vocally. Participants at approximately 90-130 sites attend each session. No participation fee is necessary.

## Methods

### Study design and participants

We used a qualitative study design with a thematic analysis approach. Qualitative methods were chosen to allow in-depth exploration of the participants’ impressions of the webinars. Recruitment of study participants was done through convenience sampling. During the spring of 2015, repeated announcements were made during the webinars and on the listserv to participating physicians located throughout the country, including primary care physicians and specialists with a special understanding in primary care, to invite them to participate in the study. Six active webinar participants, all physicians, agreed to participate. Five were in primary care, and one was a psychiatrist affiliated with a university. Five participants were male, and one was female. Two of the participants had prior experience with making presentations in the webinars. Their demographics are shown in Table 1. The Institutional Review Board approved this research at Sapporo Medical University. Written informed consent was obtained from all participants in advance.

### Data collection

A focus group (FG) was conducted to explore how the participants and their characteristic features have perceived the webinars. We chose an FG because we felt some participants’ ideas could stimulate others to expand their ideas. It was conducted on June 13, 2015, in the city of Tsukuba, where an annual meeting for the Japan Primary Care Association was being held. We took the opportunity to hold the FG at this venue because we believed a considerable number of the webinar participants were expected to attend the meeting and this provided us with an opportunity to recruit volunteers to participate in the study. The FG was conducted in the afternoon, when sessions of the annual meeting were being held. The FG was conducted in a semi-structured format. Participants were asked to answer the following questions: 1) What led you to participate in the webinars? 2) What are the webinars’ strengths and weaknesses? 3) Will you share with us, if any, episodes in which the webinars were useful or influenced you to change your practice? 4) What are the webinars’ advantages and disadvantages of being on the Internet? HO, the second author and a medical education researcher, served as the facilitator. The FG was conducted for 50 minutes and was audio-recorded and transcribed.

**Table 1:** Focus group participants

Participant	Clinical experience (years)	Gender	Years of participation in the webinars	Speaker experience	Specialty	Current workplace	Community population	Position
#1	13	Male	1	No	FM	Hospital	33000	Division head
#2	22	Male	6	Yes	FM	Hospital	8000	Director
#3	3	Male	2	No	GIM/FM	Hospital	79000	Resident
#4	21	Male	4	No	Cardiology/IM	Hospital	7000	Director
#5	13	Female	11	Yes	Psychiatry	University	186000	Assistant professor
#6	11	Male	7	No	FM	Hospital	16000	Division head

Key: FM = Family Medicine, GIM = General Internal Medicine, IM = Internal Medicine

### Data analysis

The focus group recording was transcribed. We read the data repeatedly and familiarized ourselves with them. The transcripts were first separated into meaningful segments of text by the first author (SK) using an analytical worksheet, and the segments were then open-coded. Next, we met repeatedly to review and examine the coding. This process was repeated to the point where no new codes emerged, i.e., until we reached inductive thematic saturation.^26^ Concepts were generated through selective coding. Finally, the extracted concepts were grouped into five categories. These steps ensured that the expectation of trustworthiness in qualitative research was achieved. We have different backgrounds: SK is a family physician deeply involved in the webinars, HO is a medical education specialist and MK is a psychiatrist/sociologist. We believe our different perspectives helped provide us with better understandings of the findings and added to the trustworthiness of the study.^27^

## Results

We extracted concepts from the FG transcripts to determine

the webinars’ characteristics and possible manners in which the webinars may have influenced the participants. The concepts were then grouped into categories, which were transformed into a conceptual model that consisted of five categories: 1) technological breakthroughs, 2) creation of learning opportunities, 3) external interaction, 4) stimulation of internal interaction and 5) the advantages and disadvantages of an increasing number of participants.

### Technological breakthroughs

Advances in ICT have contributed significantly to the development of the webinars. This category has three subcategories. Feedback from participants seen below is displayed in italics. (Comments have been translated from the original Japanese into English.)

### Accessible archives allowing easy review of the previous contents

Some FG participants valued 24/7 access to the archives of past handouts and the availability of DVDs of the past sessions upon request. 

“For example, when the season for childhood immunization arrived, there was a lecture on vaccinations in the PCLS…. and you can download the handout at a later time if need be and review it. This is a great plus of the PCLS. It is not just watching the lecture once and for all”. (Participant 4, male, cardiology/internal medicine, 21 years’ experience)

“… they used to distribute a free DVD of the sessions”. (Participant 5, female, psychiatry, 13 years’ experience)

“If I remember correctly, it was a [free] rental [of DVDs of past lectures]”. (Participant 2, male, family medicine, 22 years’ experience)

These FG participants stressed the usefulness of the accessibility to the webinars’ archives thanks to advances in ICT.

### Location being no barrier

The webinar participants can participate in the webinars as long as they have a personal computer and access to the Internet, which makes location no longer a barrier.

“All we do is start Safari [a browser], access the website of the Department, and put in the ID and the password. It’s stress-free”. (Participant 2, male, family medicine, 22 years’ experience)

“… I’m truly grateful about us being able to participate in the PCLS/PCC. … [N]ow one can participate no matter which hospital you work at, probably, if there is an environment that allows it.… If you can protect your time in the mornings, you can continue to participate wherever you go. I am so thankful for the learning environment that they create”. (Participant 3, male, general internal medicine/family medicine, 3 years’ experience)

As seen from their comments, the participants appreciated this accessibility.

### No extra investment needed to participate

With very few exceptions, primary care physicians have access to computers and the Internet. They can, therefore, participate in the webinars without having to buy equipment save for a web camera and a microphone for when they are the webinar’s main speaker.

“… starting from the software just before the current one, we have been able to use a Mac”. (Participant 2, male, family medicine, 22 years’ experience)

“Before that time [the change in the webinar software], I had to run around looking for a Windows machine”. (Participant 5, female, psychiatry, 13 years’ experience)

FG participants noted this ability to use personal computers in common platforms.

### Creation of learning opportunities

The webinars have created and provided comfortable, practical learning opportunities for CPD, incorporated into participants’ routines, with no commercial bias. This category includes eight subcategories.

### Regular webinars as participants’ routine

Because the webinars are held twice a week throughout the year, it has become a part of the participants’ weekly routine, similar to watching their favorite television programs.

“It starts at 7:30 AM…. The good thing is, the sessions end at 8 AM and no later. If the session goes longer, it might interfere with our daily routines, but since they end at eight, we are done, and we can go on with our work”. (Participant 2, male, family medicine, 22 years’ experience)

“… it ends at 8:05 at the latest. Everybody knows [that it is supposed to end at 8 AM], and they leave the session on their own once it hits eight, saying, “Thank you very much” in the chat box, so we all get out at just about eight”. (Participant 2, male, family medicine, 22 years’ experience)

“The tradition to report the number of participants at each site at the end of the session creates an atmosphere that the session is ending soon”. (Participant 6, male, family medicine, 11 years’ experience)

As seen above, FG participants reported being accustomed to the regular hours and predictability of the webinars.

### A comfortable learning climate

The webinars cultivated and maintained a sound and comfortable learning climate without any brutal words or harsh criticisms.

“There is nobody who monopolizes the time by asking out-of-place question after question, like in an SNS [Social Networking Service]. Everybody is well mannered, maybe because they’re grown-ups with the same cause. It feels comfortable there, and that’s one of the reasons why I am glad to be a speaker”. (Participant 5, female, psychiatry, 13 years’ experience)

The comment above exemplifies how participants felt a comfortable environment could positively affect the main webinar speaker.

### Regional contents included

In the webinars, local topics from many regions are presented at various times, providing a good balance with topics of more national scope.

“It used to cover more regional topics like horse-related trauma, poisonous mushrooms, [topics] especially [relevant to] those in Hokkaido, and they emphasized hypothermia, etc., I think. That kind of policy, the PCLS having been started at Sapporo Medical University, seems very rational. When the webinars went national thanks to the Internet, and had, like, 100 participating sites, the contents came to be chosen according to what’s common nationally or even internationally, whether it’s good or bad. But then there was a session on a regional topic … maybe the webinars cover a combination of local and national topics, and that’s an advantage of the Internet”. (Participant 6, male, family medicine, 11 years’ experience)

“Local content is also important. It is important for residents and also us to learn that there is a regional uniqueness in medicine”. (Participant 5, female, psychiatry, 13 years’ experience)

The FG participants recognized the importance of incorporating these local topics and noted their enjoyment of a mixture of these with universal topics.

### Immediate applicability to practice

The knowledge and skills that participants learned in the webinars were immediately applied to real-life situations, indicating that the webinars are changing the participants’ practice.

“Yes, the webinars have changed our practice. They cover a wide variety of topics and diseases, especially PCC. Patients similar to the ones presented often do come to the outpatient department. So we got into the habit of actively looking for them”. (Participant 1, male, family medicine, 13 years’ experience)

“For example, I wasn’t quite familiar with Bornholm disease. I’d been at the kind of level like, ‘Yeah, I’ve heard about it’. But after I heard about it on the PCLS, I was like, ‘Now I know what it is’”. (Participant 6, male, family medicine, 11 years’ experience)

“Because speakers are mostly family physicians like us, we are likely to encounter similar patients”. (Participant 6, male, family medicine, 11 years’ experience)

“We run into patients that we do not see too many of, you know. In the PCLS, they cover various topics, like pediatric or psychiatric stuff, gyne stuff, often in a pretty timely fashion. It gives us some background knowledge on various diseases that’s necessary for consulting specialists. I think it’s great that they cover a wide variety of topics in a very informative way”.  (Participant 4, male, cardiology/internal medicine, 21 years’ experience)

The practical coverage of the webinars was perceived to be useful in their daily practice by the FG participants.

### No commercial bias

With no commercial body sponsoring the webinars, it is a significant advantage for the webinar participants not having to worry about commercial bias or at least the potential for it.

“Since there is no commercial sponsor and there is no need for the speaker to connect the contents to a specific drug, the webinars are closer to real-world practice. If there were a sponsor, that would be difficult…. In a conference held by the local medical association, it is taken for granted that they do have a sponsor and, it’s like, ‘the topic is this. [The topics are limited]’”. (Participant 6, male, family medicine, 11 years’ experience)

“The advantage of these webinars is the lack of commercial bias. A beautifully done seminar spending lots of money is bound to have a commercial sponsor, and a pharmaceutical company may be staying behind the scene, so there’s got to be some bias. And to have to think that way is already a nuisance itself. I think that this type of education [the PCLS and PCC] needs to be promoted more and grow more”. (Participant 5, female, psychiatry, 13 years’ experience)

The comments above illustrate how keenly aware and appreciative some FG participants were of the absence of commercial sponsorship.

### Physicians participating of free will

Because of the ease of participation, the FG participants felt that it was up to each participant whether to participate in the webinars and in the individual sessions.

“… I guess now you can participate from any hospital as long as you have a will to participate and an environment for it”. (Participant 3, male, general internal medicine/family medicine, 3 years’ experience)

The comment above indicates participants’ belief that the ease with which to participate was at a level where such participation depended on one’s motivation.

### Sharing experiential knowledge

The practical experiences of physicians in the communities have been shared as experiential knowledge in the webinars.

“The lectures cover a wide variety of topics. And the best feature, I think, is that we can listen to the voices of doctors in the real world. It’s very interesting to me that we and the speakers have so much in common; like we all are doing the same thing, or, like, oh, ‘We had the exact same experience recently!’ [and] stuff like that”. (Participant 2, male, family medicine, 22 years’ experience)

The comment above shows that the participants believed webinars provided precious opportunities to listen to real-world experiences from elsewhere and they appreciated the fact that they often shared similar experiences.

### Learning by becoming a speaker

In the webinars, many participating primary care physicians and their specialist colleagues become speakers. It was the FG participants’ common understanding that, although there is some pressure to give a persuasive presentation, the speakers gain experience and learn new information themselves from teaching.

“… as you become more competent, you start to be a speaker”. (Participant 5, female, psychiatry, 13 years’ experience)

“I am afraid to get an email from the coordinator, asking me to be a speaker. When I run into him at a national conference or something, I go, ‘Uh-oh, there he is … What if I get asked [to give a talk]?’ I get scared, you know”. (Participant 2, male, family medicine, 22 years’ experience)

“We probably would not feel much stress if and when it is understood by all the participants that we have our share of presenting every once in a while, and we learn together by doing so. But when a newcomer comes in and feels that there are lectures of various kinds and quality, it might appear to them that it is a disadvantage [to have a participant become a speaker]”. (Participant 6, male, family medicine, 11 years’ experience)

“You learn the most when you make a presentation yourself.… And, it gives you confidence as well”. (Participant 5, female, psychiatry, 13 years’ experience)

These quotes show how the participants understood the importance and value of making a presentation for their own growth and contribution to the webinars while at the same time acknowledge feeling some stress associated with making presentations themselves.

### External interaction

The chat system plays a key role in external interaction, making such interaction easier. The participants are stimulated by questions and comments posted to the chat system by other participants. Through learning together and interaction, the participants develop a sense of being connected. This category includes three subcategories.

### Text chats making the webinars interactive

The FG participants appreciated the value of the chat system because it greatly lowered the threshold for interaction.

“I think the chat function is very useful…. I strongly feel that they have created an environment where it’s easy for us lowly youngsters to ask questions or give comments…. I am thankful that we can give comments without reservation”. (Participant 3, male, general internal medicine/family medicine, 3 Years’ experience)

“I think the chat function is so wonderful! … It makes the webinars interactive, like, you can ask other participants what they do at their place…. It makes participation not just watching and listening; you can go into it, and that’s great”. (Participant 3, male, general internal medicine/family medicine, 3 years’ experience)

The comments above reflect how well perceived and utilized the chat system is, making it easy for even young participants to ask questions or give comments.

### Learning from questions and comments posted to the chat system

The FG participants reported about having been stimulated by pointed questions or succinct comments in the chat system from a few leading participants.

“I look at the text chats and say, ‘Ah, these [astute] guys dig into that point!’” (Participant 2, male, family medicine, 22 years’ experience)

“We learn from looking at sharp questions from famous doctors, too”. (Participant 3, male, general internal medicine/family medicine, 3 years’ experience)

These and other similar comments indicate that the participants believed they could learn from other participants’ questions or comments that are sent to the speaker through the chat system.

### Virtual collegiality among the participants

The participants in the webinars have come to develop a sense of collegiality and camaraderie.

“I think that, as it was aimed in the beginning of the PCLS, it is being offered so that doctors who are in remote areas, or who are by themselves will not feel lonely. I think that that philosophy is still alive, [and] that it is one of the hidden advantages of participating in the PCLS, to be able to have a sense of connectedness or camaraderie.… Being connected and being able to ask about something, not just learning something practical, gives us a little bit of courage, I think”. (Participant 5, female, psychiatry, 13 years’ experience)

“I was surprised when a doctor in Kawasaki said to me, ‘I saw your recent lecture’. I was like, ‘How come this doctor knows that I was speaking? I wasn’t aware’’. (Participant 5, female, psychiatry, 13 years’ experience)

As noted in the comments above, the participants felt having a sense of connectedness is another highly valued feature of the webinars.

### Stimulation of internal interaction

The FG participants reported about real-time internal interaction occurring within participating sites and how they gained teaching opportunities. This category includes two subcategories.

### Issuing one ID per site eliciting on-site interaction

The setup in which participants at each site gather in one room and participate together has given rise to on-site, spontaneous discussions during the webinars, a result not originally intended by the organizers.

“The one-ID-for-one-site policy is a necessary system, although I sometimes feel it’s inconvenient. I take it positively, though. I guess it is now technically possible to give one ID for each individual, but in the beginning, because there weren’t enough accounts available, the organizers wanted doctors at each site to participate as a group. For example, in my hospital, we have about five or seven participants each time. We sometimes go into a discussion during the session and we go, “We ought to put our thoughts in the chat box.” By participating as a group, these conferences create opportunities at each site to learn”. (Participant 6, male, family medicine, 11 years’ experience)

As seen from this comment, real-time internal interaction in the form of spontaneous discussion can occur within participating sites without interrupting the sessions.

### Creating opportunities for teaching and having students see physicians learn

The FG participants appreciated the value of having opportunities to teach students and to show students and residents part of their life-long learning modalities and how to keep up-to-date.

“When students come for a week-long clerkship, we have them participate in the webinars although they don’t get to participate longitudinally. It’s to show them that there is such a tool you can keep up-to-date with, I mean, a way to learn not face-to-face. I don’t know if they find the content interesting or not, but at any rate, we say, “This is what we do, and we want you to take a look” and have them come at 7:30 in the morning”. (Participant 6, male, family medicine, 11 years’ experience)

“We are practicing in a rural community, and, when med students come for a clerkship, we can show them that we are practicing at the standard, national level although we are such a long way from the big cities”. (Participant 4, male, cardiology/internal medicine, 21 years’ experience)

This comment indicates that the FG participants believed the webinars help show students that there are opportunities to keep up-to-date even in rural areas and that the doctors are learning new information and skills themselves.

### Advantages and disadvantages of nationwide expansion

The increase in the number of sites that participate in the webinars was thought to have advantages and disadvantages. This category includes three subcategories.

### Higher quality achieved through nationwide expansion

With participants initially limited to Hokkaido, the webinars used to enjoy local popularity. The nationwide expansion of the membership led to diversity in participants and content as well as continuous improvements, which have led to higher quality.

“I feel that it’s good for the graduates of the local medical school in my area and its students to be able to listen to the talks from outside the region, from all over Japan, that is. I do not know how they are taking it, whether they sense a difference from what they do there. But I kind of feel that we are being able to listen to fairly uniform talks at the national level”. (Participant 1, male, family medicine, 13 years’ experience)

“Maybe it was good that the webinars continued to expand its membership. If we had limited the participants to just ourselves in Hokkaido, the content might have deteriorated to lower-level stuff. It may be that we were able to maintain quality because we had lots of people listening [from all over Japan]”. (Participant 5, female, psychiatry, 13 years’ experience)

Comments such as these indicate that the FG participants felt the webinars’ nationwide expansion helps maintain the quality of both the participants’ standard and the webinars themselves.

### Speakers become more nervous

Although the more significant number of participating sites was generally taken positively, some FG participants shared their fear of having to speak to a broader audience than before. Moreover, no longer being able to see the faces of all participants was considered a disadvantage.

“To speak in a webinar is difficult because you do not see the reactions of your audience. I’ve got used to it, but initially I got so nervous. It was better when the software showed a picture of each site, which we no longer have. I can’t forget how nervous I was”. (Participant 5, female, psychiatry, 13 years’ experience)

This comment indicates that even an experienced speaker can feel uneasy.

### Participants concerned about an increased burden on the organizers

The FG participants, although they hoped that the webinars would grow further, shared their concern about the burden on the administration. One FG participant suggested that additional funding might ease the burden on the organizers and the moderators.

“The downside is that the workload of the moderators and the coordinator is too much.… We participants can’t think of any disadvantages in the current system. I wonder if they can continue to hold with the current manpower and the way they run the webinars”. (Participant 6, male, family medicine, 11 years’ experience)

“If there is anything to be improved about the PCLS and PCC, it would be funding. The content is just not available elsewhere.… So, it is kind of too bad”. (Participant 5, female, psychiatry, 13 years’ experience)

In the comments seen above, the participants expressed concern that the sheer workload might be becoming excessive for the moderators and organizers and suggested that increased funding could alleviate the situation.

## Discussion

To our knowledge, this is the first study that investigates over an extended period how webinars primarily designed to assist their participants view primary care physicians. As discussed above, how participants viewed these webinars, and their characteristic features were categorized into five categories: 1) technological breakthroughs, 2) creation of learning opportunities, 3) external interaction, 4) stimulation of internal interaction and 5) the advantages and disadvantages of nationwide expansion.

Technological breakthroughs played a fundamental role in the development of the webinars. In the 1990s, faster telephone lines (ISDN lines) and an expensive, dedicated terminal at each site were used to hold video conferences. As a result, video conferences tended to be costly.^5^ At present, having a personal computer and Internet access is almost the norm for physicians, which makes it possible for almost anyone to participate in the webinars easily with no added cost regardless of their location, even in very remote areas. It has also become easy to develop and maintain an online archive of the past handouts of webinars that are accessible to participants at any time. These benefits from the advances in ICT formed a firm basis upon which these webinars have been designed as discussed below.

In our study, the webinars were found to be creating learning opportunities for and influencing the participants by 1) meeting regularly, 2) providing a comfortable environment, 3) including regional contents, 4) extending their applicability to practice, 5) having no commercial bias, 6) eliciting spontaneous participation and 7) instilling an atmosphere in which participants could share experiential knowledge. In adult learning, the importance of a learning environment has been emphasized.^28^ It plays as great a role in learning as the content itself; the learner needs to feel safe, and to feel that the content is relevant to what he/she needs or will need. When the learning is relevant, it leads to motivation. In the webinars studied, the FG participants were comfortable, feeling that the learning was relevant because of their practicality and lack of commercial bias. This resulted in enhancing their motivation and continued participation. The webinars’ design closely follows the basis upon which learning occurs according to adult learning theories and underscores their importance.

Interaction plays an extremely important role in learning. Mere didactic lectures have not been shown to change physicians’ practice.^29^ One of the unique features in the webinars is the extensive and effective utilization of the chat system during the sessions. In the literature, the most beneficial aspects of videoconferencing are interacting and sharing problems with colleagues.^6^ We found that the chat system plays a key role in interaction and information sharing in these webinars. The participants liberally post questions and comments to the chat box during the session. This is in sharp contrast to the general chat system in online classes in distance education, which merely “complements the more useful asynchronous communications in forums and bulletin boards with the experience of exchanging ideas spontaneously”.^8^

We identified three important features in utilizing the chat system. First, the chat system appears to reduce the psychological barrier to expressing oneself. With text chats, the participants seem to have a lower anxiety threshold for asking questions or providing comments than raising their hands or speaking in front of others in face-to-face meetings. This may mainly be true for shy, reserved participants. Using text chats during the session does not interfere with the flow of the presentation and allows the speakers to respond at their leisure; one can choose to respond immediately or later in the session. Second, participants are stimulated by other participants through the text chats that are written during the sessions. They learn from various participants’ opinions and questions, what others usually do (regarding diagnosis and treatment) in practice and what others might do in a given situation. They are also presented with the current standard practice. They compare their level of knowledge and practice to their virtual colleagues participating in the webinars. The participants highly value this type of learning. Third, participants spontaneously interact with other participants through text chats, which is a phenomenon that has been described in distance learning as learner-learner interaction.^8^ This study is the first to describe interaction as a function of text chats in webinars in the arena of CPD in health professional education. In this context, the webinars may surpass opportunities provided by face-to-face learning by making interactive learning possible between both the speaker and the participants and among the participants themselves, both on a nationwide scale.

The second and third features were not originally intended or expected. Merely having the chat system available does not lead to effective use, however; the moderator’s role is crucial in eliciting, allowing and controlling interaction, which has been indicated in previous research.^30^ We believe that text chats have great potential, even in non-online situations, in that they may be applied to “real” lectures or conferences to lower the anxiety threshold for asking questions or providing comments, which will allow even shy learners who do not like to speak out to engage fully. This is an aspect of the webinars that needs to be pursued in future research.

Another noteworthy feature of the webinars is that when two or more members participate at a given site, interaction occurs within site. This has been discussed in distance education, but this is the first study that describes the internal interaction in the arena of online CPD for physicians.^8^ In the webinars studied, an ID and a password are issued per site, not per individual, which enables primary care physicians to gather in a room and participate together, viewing the same screen. When they do so, they often engage in spontaneous discussions and ask questions or give comments in the chat box. This type of interactive learning is unique to online conferences. Unlike in “real” seminars, participants’ voices cannot be heard by the speaker, which makes it easier for participants to interact among themselves. We believe that this is another major advantage of webinars over “real” seminars.

The teaching that can occur within the participating site also demonstrates to students that doctors continue to learn new information. The primary care physicians have the students participate in the webinars with them, which creates opportunities for teaching. Physicians also gain opportunities to show students that learning is a never-ending process. Each teacher’s actions, attitudes, and interest affect the learners indirectly, and the subliminal message is enormous. That these webinars create, these opportunities were another unexpected finding in our research.

The webinars’ expansion beyond Hokkaido has added to their value by making more interaction possible nationwide, increasing diversity among the speakers and content and reportedly achieving overall higher quality. However, there have been disadvantages. Because of the psychological pressure of speaking to a larger audience, the speakers tend to become more nervous compared with when speaking to a more local audience, and thus there has been an increase in the anxiety threshold to make a presentation in the webinars. However, because the speakers do not see the 300+ faces of the audience and because there is interaction through the chat system during the session, making a presentation in the webinars may not cause the speakers to become as nervous as they might in face-to-face seminars of similar size. This requires further research. Concerns have additionally been raised about costs and the increased burden on the organizers, which has always been an issue in sustaining the webinars. Measures to reduce the organizers’ amount of work need to be seriously considered.

### Limitations

This study has several limitations. First, the FG analysis had a small convenience sample of participants who volunteered to participate. It is possible that only those who had a more positive image and recollection of the webinars were represented; the data gathered therefore may not necessarily represent the full spectrum of opinions of those who have participated in the webinars. Moreover, the FG participants were working in metropolitan, urban or rural communities and based in hospitals or universities. We did not have any clinic-based physicians or physicians from very small communities who participated. Although a significant portion of primary care is handled by hospital-based physicians in Japan, with no clinic-based physicians in the sample, it is difficult to maintain that it was representative of all primary care physicians. Including those who discontinued participating in the webinars in the sample may have led to additional insights and should be a consideration for future research. Second, SK, who initiated the webinars and is the current coordinator, took part in the data interpretation and analysis. This could have led to potential bias. However, HO and MK were also deeply involved in the data interpretation and analysis, and HO has never attended the webinars studied, while MK only attended on a few occasions as a medical student. We believe that this triangulation prevented biased analyses of the data. Third, because the participants were physicians, we could not explore the perspectives of other healthcare professionals and medical students who participated in the webinars.

In addition to the limitations noted above, this study did not demonstrate another possible, important role of webinars: providing a space where physicians could share their feelings of uncertainty. Addressing uncertainty is part of primary care because patients present with symptoms and signs and not with a diagnosis. Primary care physicians must effectively cope with uncertain feelings and the related stress in their practice. Reflection and peer group discussions are methods for addressing uncertainty, but physicians with only a small number of colleagues or no colleagues at all may not have these opportunities.^31^ It may be beneficial for future studies to examine whether the stress due to uncertainty is relieved through sharing these feelings of uncertainty in webinars and how connectedness/camaraderie influences that process.

## Conclusion

The webinars studied are online CPD programs that were originally developed for rural primary care physicians in Hokkaido and have expanded nationwide over the last 14 years. Conducted by and for primary care physicians in Japan, the webinars have successfully created comfortable and practical learning environments. The chat system has been found to be utilized effectively to allow for interaction between sites, making it easy to ask questions and exchange information, thus increasing participants’ feelings of connection and reassurance. The use of the chat system shows enormous value in that it makes even didactic lectures interactive and turns them into good learning opportunities. It seems to surpass face-to-face learning opportunities in this regard. The webinars also generate opportunities for interaction within the sites and for teaching and showing students that primary care physicians continue to learn in order to keep their skills and knowledge up-to-date. Future research will be helpful to determine whether the webinars can help primary care physicians cope with uncertainties that they encounter in their daily practice.

### Acknowledgments

We thank the participants for their participation. SK extends his gratitude to Professor Wari Yamamoto and Professor Haruyuki Tatsumi for their continued encouragement and support, Dr. Kazuo Yagita, Dr. Mitsuharu Yoshino, Dr. Yoshinobu Fujito, Dr. Toshihiko Natsume and Dr. Shinya Aoki for their unselfish dedication to the implementation of the PCLS/PCC and all the past and present coordinators, speakers and participants for their contributions to the PCLS/PCC.

### Conflict of Interest

The authors declare that they have no conflict of interest.
